# Solid pseudopapillary neoplasm of the pancreas in two male patients: gender does not matter

**DOI:** 10.11604/pamj.2017.27.283.9800

**Published:** 2017-08-21

**Authors:** Faten Limaiem, Hafedh Mestiri, Saloua Mejri, Ahlem Lahmar, Sabeh Mzabi

**Affiliations:** 1 University of Tunis El Manar, Tunis Faculty of Medicine, Tunis, Tunisi

**Keywords:** Solid pseudopapillary, neoplasm, pancreas, male

## Abstract

Solid pseudopapillary tumour (SPT) is an unusual pancreatic neoplasm which predominantly affects young women. Less than 10% of patients with SPT in the reported literature were male. In this paper, the authors report two new cases of SPT that occurred in two male patients aged respectively 25 and 20 years old. Abdominal computed tomography scan showed a well-defined heterogeneous mass involving respectively the tail and the body of the pancreas with peripheral calcifications in the first case. The two patients underwent distal splenopancreatectomy. Histopathological examination of the surgical specimen coupled with immunohistochemical study was compatible with solid pseudopapillary tumour. On postoperative day 8, the first patient developed abdominal wall abscess and peritoneal collection. Postoperative course was uneventful for the second patient. In summary, a large, well-encapsulated cystic mass in the pancreas of a young man should raise suspicion of solid pseudopapillary tumour.

## Introduction

Solid pseudopapillary tumour (SPT) of the pancreas is an uncommon neoplasm accounting for 0,13-2,7% of all exocrine pancreatic tumours [1]. It is well known for its indolent behaviour and favourable prognosis with a predilection for young women. Less than 10% of patients with SPT in the reported literature were male [2]. With the widespread availability of high-quality imaging systems and a better understanding of its pathology, the number of cases of SPT reported in the literature has been steadily increasing [3]. In this paper, we report two new cases of SPT that occurred in male patients. Over the last seven-year period, four cases of SPT were diagnosed at the pathology department of Mongi Slim Hospital. They included two female and two male patients with a sex-ratio (M/F) = 1. The aim of the present study was to highlight the clinicopathological features of this relatively rare neoplasm with review of the current literature.

## Patient and observation

### Case 1

**Figure 1:**
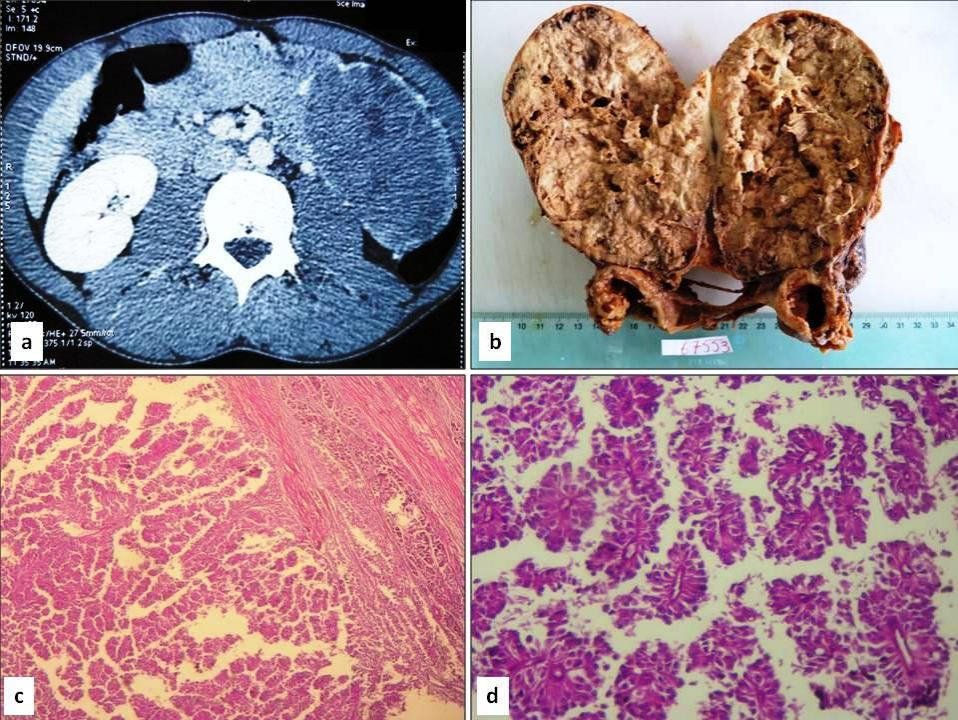
Radiological and pathological characteristics of solid pseudopapillary neoplasm in case 1. A) CT scan demonstrating a well-delineated and heterogeneous enhancing mass of the pancreatic tail with peripheral calcifications; B) cut section of the tumour demonstrating a heterogeneous predominantly solid mass surrounded by a thick fibrous capsule with extensive necrosis; C) tumour proliferation surrounded by a thick fibrous capsule. The growth pattern is heterogeneous, with a combination of soild and pseudopapillary in various proportions (H & E, original magnification x 40); D) loosely cohesive tumour cells radiating around blood vessels reminiscent of pseudorosettes in ependymoma (H & E, original magnification x 200)

A 25-year-old previously healthy male patient, presented with a five-month history of abdominal pain and slowly enlarged, palpable, non-tender upper abdominal mass. Physical examination of the patient disclosed a firm abdominal mass. Abdominal computed tomography (CT) scan showed a well-defined heterogeneous mass involving the pancreatic tail with peripheral calcifications (Figure 1a). Laboratory examinations demonstrated normal levels of pancreatic enzymes and normal liver function test results. The serum tumour markers were not performed. The patient underwent distal splenopancreatectomy. Macroscopically, the pancreatic tumour was encapsulated measuring 14 x 7 cm. On cut section, it showed mainly solid and partially cystic components and demonstrated focal haemorrhage and central necrosis (Figure 1b). Histologically, the tumour was circumscribed by a thick fibrous capsule (Figure 1c). The growth pattern of the tumour was heterogeneous with a combination of soild, pseudopapillary and haemorrhagic-necrotic pseudocystic structures. The solid areas were composed of poorly cohesive monomorphic cells that were admixed with hyalinized stromal bands containing thin-walled blood vessels surrounded by loosely cohesive tumour cells radiating around blood vessels reminiscent of pseudorosettes in ependymoma (Figure 1d). Cholesterol clefts as well as clusters of foamy histiocytes were focally noted. Immunohistochemically, tumour cells showed positive immunostaining with CD56, CD10, β-catenin, progesterone receptor and synaptophysin focally. They were negative for chromogranin and cytokeratin. The final pathological diagnosis was SPT. No adjuvant therapy was administered. On postoperative day 8, the patient developed abscess of the abdominal wall and peritoneal collection.

### Case 2

**Figure 2:**
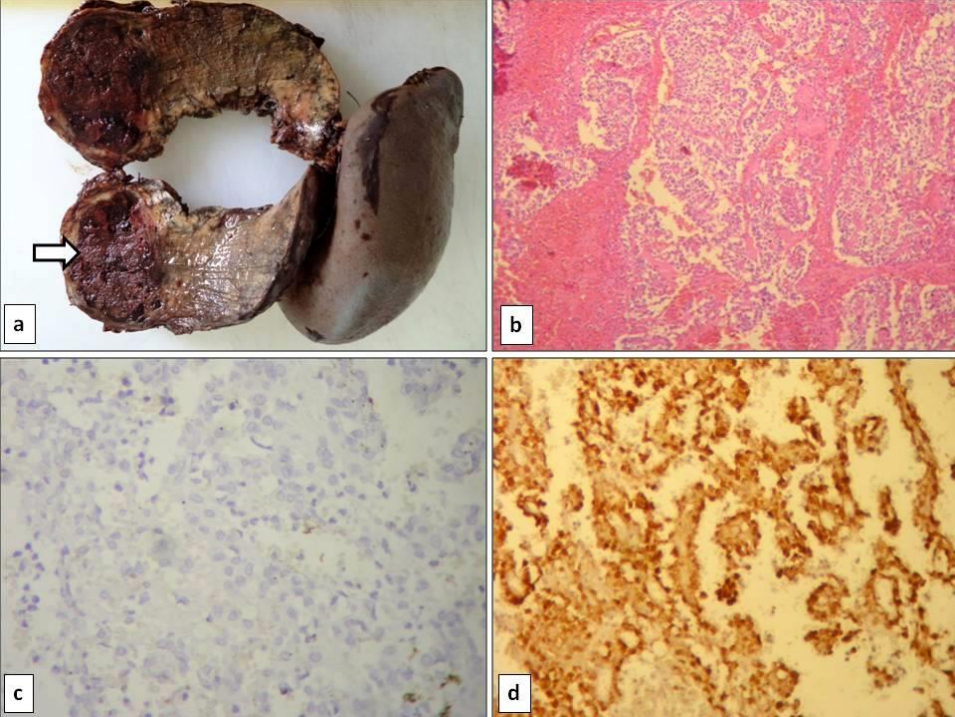
Pathological and immunohistochemical profile of solid pseudopapillary neoplasm in case 2. A) cut section of the tumour of the head of the pancreas demonstrating a heterogeneous cystic mass surrounded by a fibrous capsule with extensive haemorrhage and necrosis; B) tumour proliferation with extensive haemorrhage and necrosis (H & E, original magnification x 40); C) tumour cells were negative for chromogranin A; D) neoplastic cells showed nuclear immunolabelling for beta-catenin (Immunohistochemistry, original magnification x 200)

A 20-year-old male patient with no particular past medical history, presented with epigastric pain for the past two months. Physical examination and laboratory tests were within normal limits. Abdominal CT scan showed a well-circumscribed cystic mass involving the body of the pancreas with heterogeneous enhancement. The patient underwent distal splenopancreatectomy. Macroscopically, the pancreatic tumour was encapsulated measuring 5 x 3,5 cm. On cut section, it showed mainly cystic and partially solid components and demonstrated diffuse haemorrhage and necrosis (Figure 2a). Histologically, the tumour was circumscribed by a thick fibrous capsule. The growth pattern of the tumour was heterogeneous with a combination of soild, pseudopapillary and haemorrhagic-necrotic pseudocystic structures (Figure 2b). The solid areas were composed of poorly cohesive tumour cells radiating around blood vessels reminiscent of pseudorosettes in ependymoma. Cholesterol clefts as well as clusters of foamy histiocytes were not identified. Immunohistochemically, tumour cells showed positive immunostaining with CD56, vimentin, NSE, β-catenin (Figure 2d), progesterone receptor. They were negative for chromogranin (Figure 2c) and synaptophysin. The final pathological diagnosis was SPT. No adjuvant therapy was administered. Postoperative course was uneventful. At present, the patient is still being followed up.

## Discussion

Solid pseudopapillary tumour of the pancreas was first described by Frantz in 1959 and was included in the World Health Organization classification in 1996. Different from other pancreatic tumours, SPTs have unique epidemiological characteristics. They mainly affect young women with a female-to-male ratio of 9,78:1 [2]. Solid pseudopapillary tumour of the pancreas are exceedingly rare in male patients. Due to their rarity, clinical data on SPTs in male patients are scarce in the literature [3]. The age at diagnosis ranges from 2 to 85 years with a mean age of 21,9 years [2]. Compared with female patients, male patients were older, with an average age of 43,1 years. Our patients were aged 25 and 20 years old respectively. The initial presentation of SPT is usually nonspecific. Upper abdominal pain is the most common symptom (46.5%), followed by a slowly enlarged, palpable, non-tender upper abdominal mass (34.8%). One of our patients presented with a five-month history of abdominal pain and slowly enlarged, palpable, non-tender upper abdominal mass and the second patient presented with epigastric pain. The most common location of the SPT is the tail (35.9%) and the head (34%) of the pancreas [2]. Accurate preoperative diagnosis of SPT is difficult because of the similarity of the findings among cystic lesions of the pancreas. On radiological examinations both a capsule and intratumoral haemorrhage are important clues to the diagnosis because they are rarely found in other pancreatic neoplasms [4]. On ultrasonography, CT scan or MRI, the lesion is usually large, its internal structure goes from cystic thick-walled or with an inner irregular margin to a predominantly solid mass with some cystic component [4]. Although some imaging characteristics are suggestive of SPT, percutaneous fine needle cytology of the cystic wall can be used to obtain a possible preoperative histological diagnosis [5]. Complete surgical removal with *en bloc* resection of the involved organ is the treatment of choice. Distal pancreatectomy with or without splenic preservation can be performed for tumours in the body or tail of the pancreas, and a classic or pylorus-preserving pancreaticoduodenectomy for tumours of the pancreatic head [6].

In contrast to conventional ductal adenocarcinomas, complete surgical resection of SPT has been reported to provide a more than 95% cure rate [6]. The majority of SPT are considered to be of low malignant potential with only 10-15% of cases being malignant, showing local infiltration, recurrence or distant metastasis [7, 8]. There are few data available in the literature regarding the differences between male and female patients. Machado et al performed a retrospective study which included 27 female patients and 7 male patients with SPT of the pancreas [9]. They reported that male patients with SPT had distinct patterns of onset and aggressiveness compared with female patients and would be best treated by more radical surgery. Macroscopically, SPT usually form large, round, solitary masses (average size : 8-10 cm), and are often fluctuant. They are well-demarcated from the surrounding pancreas and may appear to be encapsulated as it was the case in our two patients [10]. Capsular or punctate calcifications are occasionally observed in SPT. The cut section reveals lobulated light brown to yellow solid areas and zones of haemorrhage, necrosis and cystic degeneration filled with necrotic debris. Histologically, SPT have a distinctive microscopic appearance. The growth pattern is heterogeneous, with a combination of soild, pseudopapillary and haemorrhagic-necrotic pseudocystic structures in various proportions. The solid areas are composed of poorly cohesive monomorphic cells that are admixed with hyalinized to myxoid stromal bands containing thin-walled blood vessels. Pseudopapillae are formed when the poorly cohesive neoplastic cells drop away, leaving variable aggregates of loosely cohesive cells between the fibrovascular stalks [10].

Degenerative features are commonly seen in SPT. Hyaline globules and microvesicles reflect cellular degeneration of the tumour. Foamy histiocytes are also seen within the tumours. Haemorrhage, cholesterol clefts and calcification are often encountered and may be prominent. Immunohistochemically, SPT are usually positive for vimentin, β-1-antitrypsin (AAT), progesterone receptor, neuron-specific enolase (NSE), CD56 and CD10. In contrast to diffuse and strong expression of vimentin, cytokeratin (CK) with a broad spectrum is usually negative or only focally positive. Among the various subtypes of CK, CK8 and CK18 are more often positive, and CK7 and CK19 are usually negative. Most SPT have active mutations of the β-catenin gene, and immunostaining for β-catenin reveals nuclear expression of this protein as it was the case in our two patients [10]. Histological indicators predicting the aggressive behaviour of SPT include capsule thickness of more than 2 mm, high nuclear grade, prominent necrobiotic nests, capsule invasion into the surrounding normal pancreatic tissue and vascular invasion [7]. Other studies indicate that DNA aneuploidy, double loss of X chromosomes, trisomy for chromosome 3, unbalanced translocation between chromosomes 13 and 17 found in SPT patients are associated with its aggressive behaviour and may be indicators of its possible metastatic potential [11, 12]. The pathogenesis of SPT still remains unknown. Many investigators believe it to arise from a multipotential primordial stem cell. Some authors reported that 62,5% of SPT patients are infected with hepatitis B virus (HBV), which can induce over-expression of β-catenin in tumour cells, indicating that HBV infection may be involved in the pathogenesis of SPT, which, however, has not been confirmed [13].

## Conclusion

In summary, a diagnosis of SPT should be considered in young men presenting with large and well-circumscribed pancreatic cystic masses. It is important for the pathologist to be familiar with its salient clinical, cytopathologic, histopathologic and immunohistochemical features. Such knowledge is essential to differentiate it from other potentially more aggressive pancreatic neoplasms.

## Competing interests

The authors declare no competing interests.
